# Asymptomatic papillary fibroelastoma of the Aortic valve in a young woman - a case report

**DOI:** 10.1186/1476-7120-7-43

**Published:** 2009-09-02

**Authors:** Fragiskos Parthenakis, Evangelia Nyktari, Alexandros Patrianakos, Antonis Pitsis, Anthoula Asimaki, Panos Vardas

**Affiliations:** 1 Cardiology Department, University Hospital of Heraklion, Crete, Greece; 2 Cardiosurgery Department, Agios Loukas, Thessaloniki, Greece

## Abstract

Echocardiography represents an invaluable diagnostic tool for the detection of intracardiac masses while simultaneously provides information about their size, location, mobility and attachment site as well as the presence and extent of any consequent hemodynamic derangement.

A 29-year-old asymptomatic young woman with incidental transthoracic echocardiographic (TTE) discovery of an aortic valve mass is presented. The 2-dimensional TTE showed a mobile, pedunculated mass, attached by a thin stalk to the aortic surface of the right coronary aortic cusp at the junction of its base with the anterior aortic wall. The importance of valve sparing tumour resection even in asymptomatic patients is emphasised.

## Background

Primary intracardiac tumours are rare with a prevalence ranging from 0.0017 to 0.28% in various autopsy series and in adults originate most frequently from the endocardium, followed by the cardiac muscle, and, most infrequently, the pericardium. [1-3] Approximately 75% of primary cardiac tumours are benign. [4] Papillary fibroelastomas account for less than 10% of all cardiac tumours, representing the most common valvular and the second most common cardiac benign tumour, following myxomas. [5-7]

However, their true incidence is largely unknown as the widespread availability of high resolution echocardiography and the implementation of new imaging modalities has made recognition of smaller and ill-defined lesions more frequent now than in the past [4,8].

Invaluable insight into the epidemiology, diagnosis, prognosis and treatment of cardiac papillary fibroelastomas has been provided by two systematic reviews. [9,10]

The clinical presentation of papillary fibroelastoma varies from asymptomatic to severe embolic complications.

We describe the case of an asymptomatic young woman with an aortic valve papillary fibroelastoma incidentally diagnosed by echocardiography and successfully managed with cardiac surgery. This case illustrates the sensitivity of echocardiography for the diagnosis of rare cardiac tumours especially when new techniques such as three dimensional echocardiography are implemented providing invaluable information on the specific echocardiographic characteristics of each tumour. This is of great importance as the presence of some low or high-risk features of the tumour can affect the prognosis and therapeutic management of the patient.

## Case report

A 29-year-old woman was referred for routine echocardiographic examination to our echocardiography laboratory as a part of preathletic screening. She was asymptomatic, with no past medical history and physical findings upon examination as well ECG recordings were unremarkable.

**Figure 1:**
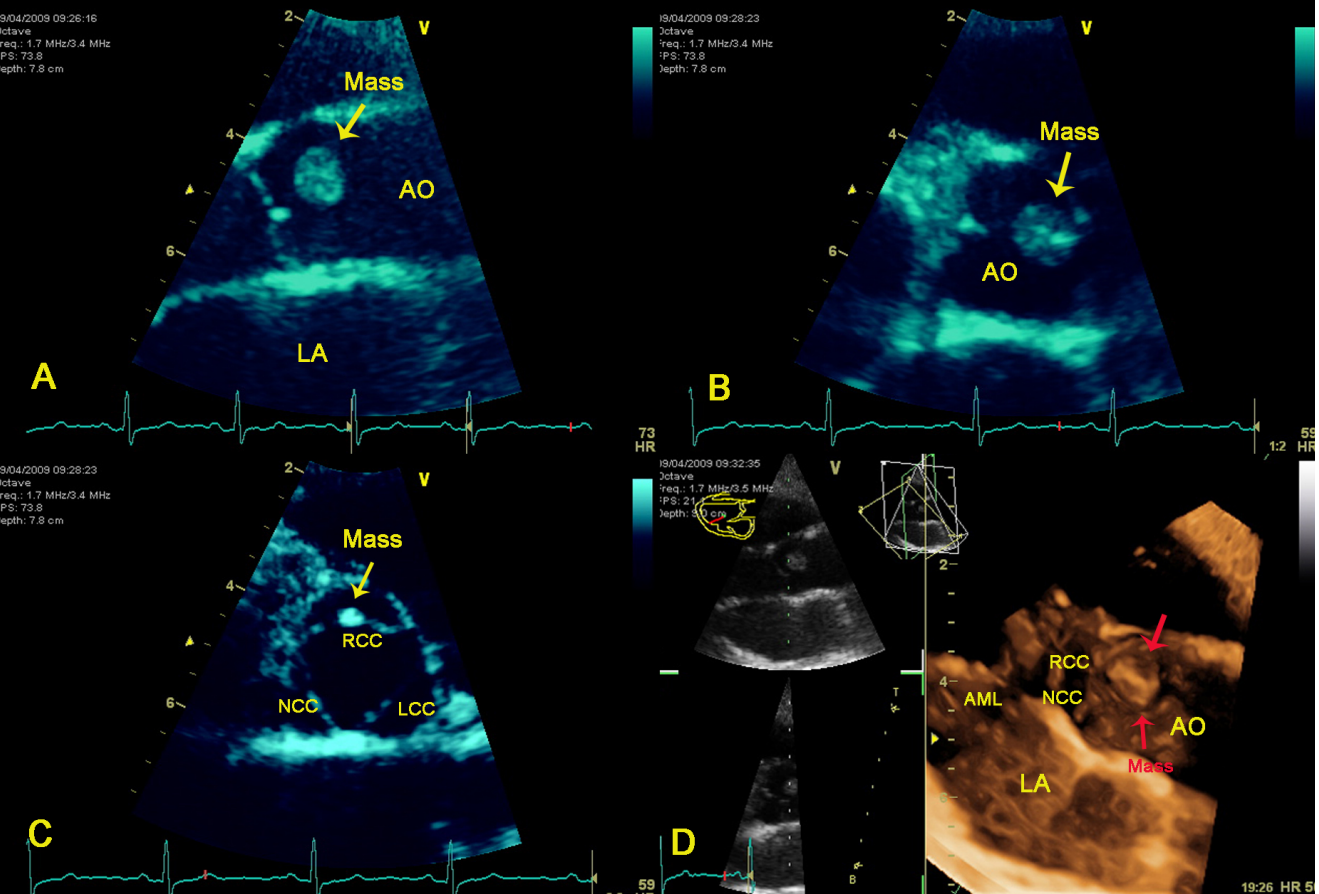
**A. Long axis view of the aortic valve. Conventional 2D-echocardiography showing a large mass originating from the aortic surface of the valve**. B. Short axis view of the aortic valve demonstrating in diastole a large papillary fibroelastoma of the right coronary cusp. C. Short axis view of the aortic valve demonstrating the right coronary cusp papillary fibroelastoma in systole. D. 3D-echocardiography depicting the origin of the tumour from the right coronary cusp. AO = Aorta, LA = Left atrial, RCC = Aortic right coronary cusp, NCC = Aortic non coronary cusp, LCC = Aortic left coronary cusp, AML = Anterior mitral leaflet.

At transthoracic echocardiography a mass 11.3 × 8.2 mm attached to the base of the right coronary cusp adjacent to the anterior aortic wall was detected. The mass was a pedunculated, mobile, round, echo dense, stipple in texture structure, with well-demarcated borders, features typical of a fibroelastoma. (Figure 1)(Additional files 1,2,3). It projected in the arterial lumen of the aorta without causing any aortic insufficiency while the rest echocardiographic examination revealed an estimated left ventricular ejection fraction of 0.65 and no other valvular abnormalities.

Because of the gross appearance and the location of the lesion a working diagnosis of papillary fibroelastoma was made. On the basis of the potential cardioembolic risk either of the mass itself or of associated thrombus and the possibility of further enlargement the patient although asymptomatic at the time of diagnosis was referred for elective surgical removal. A valve sparing technique with simple shave excision of the tumour was undertaken with particular care in avoiding embolization and ensuring that no remnants from fragmentation of this friable tumour were left behind both locally on the cusp and in the vicinity of ascending aorta and left ventricle.

**Figure 2:**
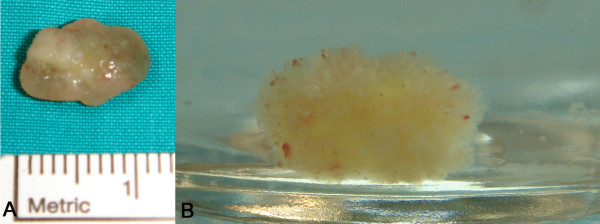
**A. Gross specimen of the mass after surgical excision demonstrating the size and the characteristic gelatinous appearance of papillary fibroelastoma**. B. Characteristic sea anemone-like appearance of papillary fibroelastoma after immersion in normal saline.

**Figure 3:**
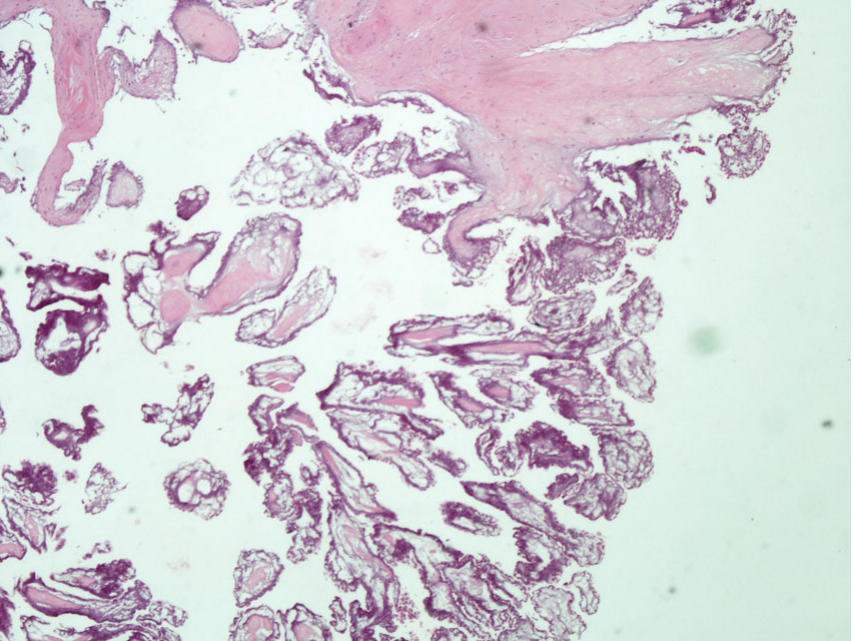
**The histological specimen in the hematoxylin & eosin stain (magnification ×40)**. A benign lesion with multiple papillary fronds is shown. Histologically, endothelium surrounds core of loose connective tissue consisting of acid mucopolysaccharide matrix, smooth cells, collagen, and elastin fibers.

The resected lesion had a flower-like appearance with frond-like projections deriving from the tumour, most pronounced after immersion in saline solution (sea-anemone picture) (Figure 2) suggesting what the histopathologic examination confirmed to be a papillary fibroelastoma (Figure 3). The patients' recovery was uneventful. A follow-up echocardiogram at 4 weeks did not demonstrate any tumour recurrence or aortic regurgitation. The patient will be followed-up in one year with repeat echo.

## Discussion

Cardiac fibroelastomas occur sporadically, exhibit a wide distribution of age, with predominance between the 4^th ^and 8^th ^decade of life and in male sex. [2,9] They are generally small, friable, slow growing tumours with multiple avascular papillary fronds made of collagen, elastic fibres and proteoglycans. Their pathogenesis remains uncertain. Their resemblance to Lambl's excrescences has led initially to the suggestion of a common origin, perhaps from trauma of the endothelium and superimposed microthrombi [5]. Furthermore, immunohistochemical studies have introduced the concept of a virus-induced local growth [11] which together with the microthrombus and valve degeneration theories further support that these are acquired rather than congenital lesions. Interestingly Kurup et al. have suggested that the procedure of open heart surgery may also cause papillary fibroelastomas and have reported that whereas non-iatrogenic tumours were likely to be single, iatrogenic tumours were likely to be multiple[12]

The majority of patients are asymptomatic and the tumours are most often incidentally diagnosed at the time of routine echocardiography, cardiac catheterization, cardiac surgery or autopsy. [2,8] Although histologically benign, papillary fibroelastomas have the potential to lead in life threatening complications from embolization of fragments into the coronary arteries, systemic circulation and pulmonary circulation depending on their size, mobility and location. Cerebral and retinal arteries are typically affected through embolization of fibrin thrombus or parts of the tumour giving genesis to the most common presenting symptoms of neurologic impairment ranging from stroke to transient ischemic attack. Papillary fibroelastomas can also cause angina, myocardial infarction or even sudden death through direct occlusion of the coronary arteries or embolization to a coronary vessel as well as heart failure and peripheral embolism. [5,9,10,13-15]Surprisingly, although commonly valvular they are rarely associated with valvular dysfunction.

The widespread use of trans-thoracic as well as transoesophageal echocardiography, has led to earlier diagnosis and treatment. Indeed, two-dimensional conventional echocardiography is the optimal non-invasive technique for imaging small masses (<1 cm) or masses arising from valves providing excellent morphologic and functional information. [3]. It provides the best spatial and temporal resolution and demonstrates an overall sensitivity of 77% rendering it first choice among the different cardiac imaging modalities [10,16]. Furthermore, with the application of dynamic three-dimensional echocardiography the precise characterisation of the tumour and its spatial relationships is allowed in terms of location, size, surface, mobility and relation to the aortic valve.

It is generally the only imaging modality required preoperatively except for the cases where the presence of coronary disease must be evaluated. Cardiac catheterization is associated with an added risk because the catheter may dislodge a fragment of the tumour and lead to embolism. [9] Patients with localization of the tumour in the aortic valve will probably need a non-invasive modality in order to evaluate the coronary anatomy and avoid the high cardioembolic risk.

Therapeutic decisions should take into consideration not only the presence of symptoms but also the potential of life threatening complications. There is a general consensus that symptomatic patients should be referred for curative surgical excision of the tumour. In addition asymptomatic patients with large (>1 cm) mobile masses especially left sided, as in our case, should also be considered candidates for curative surgical excision due to the increased risk of cardiovascular complications from embolization and sudden cardiac death[17] On the other hand, asymptomatic patients with small left sided non-mobile lesions are being closely followed up with echocardiography until symptoms develop or tumours enlarge and become mobile. [9,10]. In this way echocardiography proves to be an invaluable tool for decision making. Patients not candidates for surgical treatment should be treated with long term anticoagulation, despite the lack of guidelines to support this strategy.

## Competing interests

The authors declare that they have no competing interests.

## Authors' contributions

FP been involved in revising it critically for important intellectual content and has performed the echocardiography; APi gave his surgery opinion and the postoperative photographs; AA performed the histological analysis and gave the photo of the specimen. AP has involved in 3 dimensional echocardiography and in revising the manuscript. FPa and EN wrote the manuscript. P. Vardas had the general supervision.

All authors read and approved the final manuscript.

## Consent

Written informed consent was obtained from the patient for publication of this case report and any accompanying images. A copy of the written consent is available for review by the Editor-in-Chief of this journal.

## Supplementary Material

Additional file 1 **Parasternal long axis view**. This is a movie clip, demonstrating the classical echocardiographic picture of the papillary fibroelastoma of the aortic valve. Click here for file

Additional file 2 **Parasternal short axis view**. Another movie clip demonstrating short axis view of the aortic valve and the right coronary cusp papillary fibroelastoma. Click here for file

Additional file 3 **Parasternal long axis view**. 3D-echocardiography depicting the origin of the tumour from the right coronary cusp. Click here for file
